# β-Caryophyllene Inhibits Oxaliplatin-Induced Peripheral Neuropathy in Mice: Role of Cannabinoid Type 2 Receptors, Oxidative Stress and Neuroinflammation

**DOI:** 10.3390/antiox12101893

**Published:** 2023-10-22

**Authors:** Jonathan Paulo Agnes, Barbara dos Santos, Raquel Nascimento das Neves, Vitória Maria Marques Luciano, Larissa Benvenutti, Fernanda Capitanio Goldoni, Roberta Giusti Schran, José Roberto Santin, Nara Lins Meira Quintão, Alfeu Zanotto-Filho

**Affiliations:** 1 Department of Pharmacology, Universidade Federal de Santa Catarina (UFSC), Florianópolis 88040-900, Brazil; jonathan.agnes@posgrad.ufsc.br (J.P.A.); barbara.santos.b@grad.ufsc.br (B.d.S.); r.neves@posgrad.ufsc.br (R.N.d.N.); vitoria.maria.marques@ufsc.br (V.M.M.L.); roberta.schran@posgrad.ufsc.br (R.G.S.); 2 Postgraduate Program in Pharmaceutical Sciences, Universidade do Vale do Itajaí (UNIVALI), Itajaí 88302-901, Brazil; larissabenvenutti@univali.br (L.B.); f238620@dac.unicamp.br (F.C.G.); jrs.santin@univali.br (J.R.S.); nara.quintao@univali.br (N.L.M.Q.)

**Keywords:** oxaliplatin, phytocannabinoids, neuropathic pain, oxidative stress, neuroinflammation

## Abstract

Peripheral neuropathy is an important adverse effect caused by some chemotherapeutic agents, including oxaliplatin (OXA). OXA-induced peripheral neuropathy (OIPN) is a challenging condition due to diagnostic complexities and a lack of effective treatment. In this study, we investigated the antiallodynic effect of β-caryophyllene (BCP), a cannabinoid type 2 (CB2) receptor agonist, in a mouse model of OIPN. BCP treatment inhibited OXA-induced mechanical and cold allodynia in both preventive and therapeutic drug treatment regimens. Experiments with the CB2 receptor agonist GW405833 confirmed the role of CB2 receptors in OIPN. The CB2 antagonist SR144528 abrogated the anti-nociceptive effect of BCP on mechanical allodynia, without impacting OXA-induced sensitivity to cold. BCP decreased neuroinflammation, as inferred from TNF, IL-1β, IL-6, and IL-10 profiling, and also reduced ROS production, lipid peroxidation, and 4-hydroxynonenal protein adduct formation in the spinal cords of OXA-treated mice. BCP did not affect the antitumor response to OXA or its impact on blood cell counts, implying that the cytotoxicity of OXA was preserved. These results underscore BCP as a candidate drug for OIPN treatment via CB2 receptor-dependent mechanisms, and anti-inflammatory and antioxidant responses in the spinal cord.

## 1. Introduction

Chemotherapy-induced peripheral neuropathy is a morbidity frequently observed in therapies with platinum compounds, taxanes, and vinca alkaloids. Peripheral neuropathy caused by chemotherapy can occur both acutely and chronically, persisting for months to years after the completion of chemotherapy [1,2]. The symptoms include hyperalgesia, paresthesia, dysesthesia, burning and/or electric shock sensations, and lancinating pain in more severe cases. These symptoms are primarily felt in the hands and feet, the so-called glove-and-stocking pattern [1,2]. Among the platinum derivatives, oxaliplatin (OXA) is particularly notable for its ability to cause acute neuropathy in up to 98% of patients [3]. When combined with other drugs, such as in the FOLFOX protocol (leucovorin, fluorouracil, and OXA) used in colorectal cancer treatment, a 71% incidence of neuropathy symptoms was observed among study participants, with 84% experiencing some degree of functional impairment or reduced quality of life up to 25 months after chemotherapy cessation [1,3,4,5].

The pathophysiology of OXA-induced peripheral neuropathy (OIPN) is multifactorial, involving the participation of calcium, potassium, and sodium channels, and alterations in the function of transient receptor potential channels, oxidative stress, and neuroinflammation among the described mechanisms [6,7,8,9,10,11]. The formation of platinum–DNA adducts is a critical factor in the development and chronicity of peripheral neuropathy by platinum compounds. Platinum binding to DNA can occur in nuclear and mitochondrial compartments, compromising neuronal cell functions and activating cell death pathways [12,13,14,15]. Additionally, the overproduction of reactive oxygen species (ROS) and pro-inflammatory cytokines (such as TNF and IL-1β) in the dorsal root ganglion and spinal cord have been associated with neuronal dysfunction and sensitivity to cold and mechanical stimuli in OIPN [8,9,16,17,18,19,20,21]. In this context, interventions that reduce neuroinflammation and ROS-induced damage may be useful for mitigating neuronal damage and painful symptoms related to OXA treatment.

Cannabinoid type-2 (CB2) receptors are key components of cannabinoid signaling, regulating important physiological and pathological conditions [22]. CB2 receptors are constitutively expressed in immune cell populations, such as B-cells, NK cells, monocytes, and neutrophils [23]. In the central nervous system, CB2 receptors are expressed in microglia [24,25,26], with contrasting results in astrocytes under physiological and pathological conditions (reviewed in [25]). The downstream effects of CB2 receptor activation include immunomodulatory, anti-inflammatory, and antioxidant actions [22,27]. Of note, CB2 receptor activation produces therapeutic responses devoid of the psychotropic effects typically observed with CB1 receptor agonists. Recently, β-caryophyllene (BCP), a natural sesquiterpene present in cannabis and non-cannabis plants, such as oregano (*Origanum vulgare* L.), cinnamon (*Cinnamomum* spp.), cloves (*Syzygium aromaticum)*, and black pepper (*Piper nigrum* L.), has received attention due to its selective agonist property against CB2 receptors [22,28,29]. BCP exerts multiple pharmacological effects, including anti-inflammatory and anti-nociceptive activities, in different disease models [22,28,29,30,31,32,33]. BCP was characterized as a CB2 receptor agonist (Ki value = 155 nM) and lacked affinity for CB1 [22,28]. In addition to classical CB2-mediated effects, it has been described that BCP modulates PPARγ receptors [31,32,34], and also exerts antioxidant-related responses [35,36,37].

In this study, we evaluated the effect of BCP treatment on nociceptive, inflammatory, and oxidative stress parameters in a mouse model of OIPN, and determined the contribution of CB2 receptors to the antiallodynic phenotype. In addition, we monitored the impact of BCP on OXA antitumor effects and hematological toxicity. The results show that BCP is a promising compound for the pharmacological control of OIPN through mechanisms involving CB2 receptor signaling, and reduced oxidative stress and inflammation in the spinal cord of OXA-treated mice.

## 2. Materials and Methods

### 2.1. Drugs

OXA for intravenous administration was purchased from Eurofarma (São Paulo, Brazil), stored at 4 °C, and dissolved in sterile water for injections just before administration, following the manufacturer’s instructions. β-Caryophyllene (PubChem CID: 20831623; IUPAC name: (1S,4E,9R)-4,11,11-trimethyl-8-methylidenebicyclo [7.2.0]undec-4-ene) was purchased from Sigma-Aldrich (#W225207), and stored at room temperature according to the manufacturer’s specifications. BCP was diluted in soybean oil immediately before administration. The CB2 antagonist SR144528 (SML1899; Sigma-Aldrich, St. Louis, MO, USA) was dissolved at 1 mg/mL in a solution of phosphate-buffered saline (PBS):DMSO:ethanol (8:1:1), and stored at −20 °C. GW405833 (G1421; Sigma-Aldrich, St. Louis, MO, USA) and GW9662 (M6191; Sigma-Aldrich, St. Louis, MO, USA) were dissolved in PBS with 10% DMSO, and stored at −20 °C. These stock solutions were thawed and diluted in 0.9% NaCl immediately before administration.

### 2.2. Ethics Statement

All experimental procedures were performed according to the guidelines of the National Institutes of Health Guide for Care and Use of Laboratory Animals, and the National Council for the Control of Animal Experimentation (CONCEA) recommendations for animal care. The research protocol was approved by the Committee for Ethics in Animal Experimentation of the Universidade Federal de Santa Catarina (CEUA-UFSC), under the protocol number 1670201021.

### 2.3. Animal Housing, Randomization, Blinding, and Humane Endpoints

Female Swiss albino mice (outbred) were obtained from our Institutional Animal Core Facility (Biotério Central, UFSC, Florianópolis, Brazil), housed in cages with sawdust bedding at a controlled room temperature of 23 ± 1 °C, under a 12 h light/dark cycle (lights on at 7 a.m.), and water and standard chow were provided ad libitum. The mice were acclimated for two weeks in the animal facility before performing any experiments. At the time of tumor implants (protocol day −7), the mice were 13 weeks old and had body weights of 35.4 ± 3.1 g (mean ± standard deviation). After the tumor implants and before the first administration of OXA, the animals were acclimated for 3 days in the experimentation room (3 h/day) and in von Frey (30 min/day), cold plate (1 min/day), and Hargreaves (1 min/day) apparatus for 2 days before baseline sensitivity tests were taken. Afterwards, the animals were randomly allocated using a randomized block design (6 animals per cage), in order to obtain groups with similar baseline withdrawal thresholds. The group size (*n* = 8/group) was calculated based on mechanical and cold sensitivity data from our previous studies [9], with a probability of type I/II error of α = 0.05 and β = 0.2 (power: 1 − β). The experiments presented in this study used 168 mice. In addition, 24 animals were used for Ehrlich ascites carcinoma culture in vivo, as described below. Humane endpoints were monitored throughout the experiments, and the animals were excluded from the study if they exhibited signs of pain or distress, such as piloerection, hematuria, bleeding, cyanosis, impaired motor coordination, decreased mobility, pain (Grimace scale), weight loss (≥20%), tumor necrosis/ulceration, and tumor volume larger than 1.5 cm^3^. Blinding/masking: A first investigator (JPA) administered the treatments and performed nociception tests based on the randomization table. Thus, the nociceptive tests were unmasked to JPA. This investigator was the only person aware of the treatment group allocation. VMML contributed with JPA by preparing treatments, and monitoring body weight and tumor growth. JPA was responsible for euthanasia, and two other investigators (BS and VMML) performed tumor weighing, blood/tissue harvesting and processing, and sample coding. LB, FCG, and RGS, unaware of the treatment, performed the biochemical assays and analyzed the results.

### 2.4. Tumor Implants

Ehrlich tumor implantation was performed following our previous studies [9]. Ehrlich ascites tumor cells were obtained from nitrogen-frozen aliquots, thawed, centrifuged, and cultured in vivo via intraperitoneal passaging (weekly) in female Swiss albino mice. After three passages, the ascites tumor cells were collected and counted, and 3 × 10^6^ viable cells in 0.1 mL of PBS were implanted into the fourth mammary glands of female Swiss mice previously anesthetized with isoflurane/oxygen. Once a tumor mass was palpable (~7 days post-implant), the tumor growth was monitored using a caliper. Tumor volume (mm^3^) was calculated according to the following formula: 0.5236 × a × b^2^, where “a” is the long axis (length) and “b” is the short axis (width) of the tumor.

### 2.5. OXA-Induced Peripheral Neuropathy and Pharmacological Treatments

**Figure 1:**
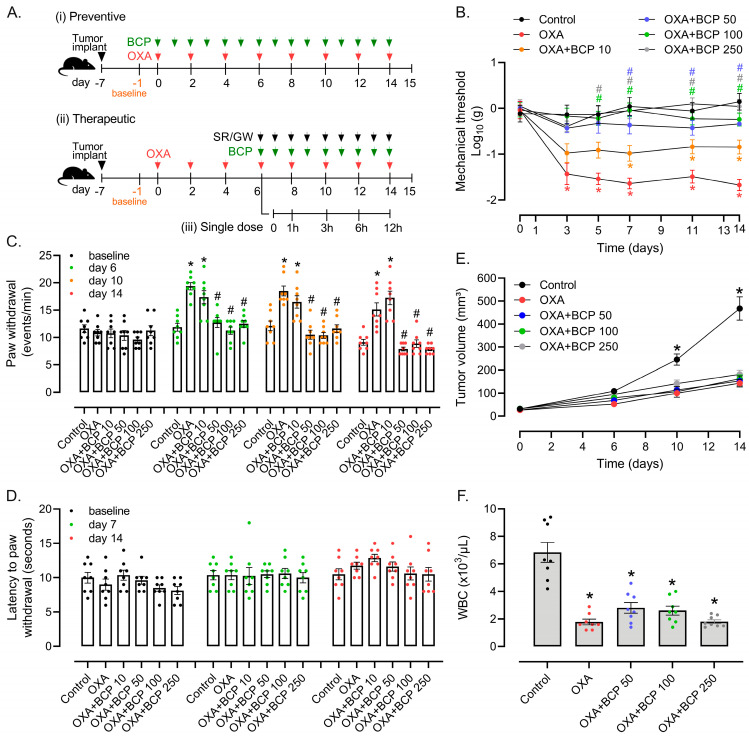
BCP inhibits mechanical and cold allodynia induced by OXA in the preventive treatment scheme. (**A**) Schematic representation of the experimental protocols performed in this study (described in the Section 2). (**B**) von Frey, (**C**) cold plate, and (**D**) Hargreaves tests; (**E**) tumor growth kinetics; and (**F**) white blood cell count (WBC) in Ehrlich tumor-implanted mice treated with OXA combined with different doses of BCP (10, 50, 100, and 250 mg/kg, 1×/day, gavage) in the preventive treatment scheme. Baseline thresholds were taken on protocol day −1, and OXA and BCP treatments started on day 0. Legend: Control (vehicle-treated); OXA (oxaliplatin); BCP “X” (β-caryophyllene; “X” represents the dose in mg/kg). * Different from the vehicle/control group in (**B**–**D**,**F**) and different from all other groups in (**E**), considering the same experimental time point; ^#^ different from the OXA group at the same experimental time point (ANOVA; *p* < 0.05, *n* = 8/group).

The OIPN protocol started once solid, measurable tumors were formed. One day before the first dose of OXA (protocol day −1), baseline sensitivity thresholds were taken. From protocol day 0 onwards, OXA (5 mg/kg) was administered intraperitoneally (i.p.), every 48 h, totaling 8 doses (protocol days 0, 2, 4, 6, 8, 10, 12, and 14), and the animals were euthanized on the 15th day of the protocol. The different drug schemes used herein are detailed below and summarized in Figure 1A. The doses of BCP, SR144528, GW405833, and GW9662 were selected from previous publications [34,38,39,40,41].

Preventive protocol: This experiment was designed to screen the pharmacological potential of BCP in preventing OXA-induced peripheral neuropathy symptoms. Ehrlich tumors were implanted on protocol day −7 and, from protocol day 0 onwards, the animals (*n* = 8/group) were treated with intraperitoneal injections of OXA (5 mg/kg) on alternate days for 14 days. BCP was given daily via gavage (200 μL/dose) from day 0 to 14, totaling 15 doses. The control group received 0.9% NaCl (i.p., on alternate days) and soybean oil (gavage, daily) as vehicle controls for OXA and BCP, respectively. The animals were euthanized on protocol day 15.

Therapeutic protocol: This experiment was designed to evaluate the effect of BCP upon an established neuropathic phenotype induced by OXA, as we previously described [9]. In the therapeutic protocol, Ehrlich tumors were implanted on protocol day −7 as described above, and baseline nociceptive thresholds were taken on day −1. From protocol day 0 onwards, the mice received OXA on alternate days, as described above. Mechanical sensitivity was monitored via von Frey tests until consistent induction of mechanical allodynia (typically after 3 doses of OXA). On protocol day 6, the animals were treated with BCP at 25, 50, or 100 mg/kg or soybean oil (BCP vehicle), 1×/day, via gavage (*n* = 8/group). The control group received 0.9% NaCl (i.p., every other day) and soybean oil (gavage, daily; from day 6 to 14) as vehicle controls for OXA and BCP, respectively. When used, the CB2 antagonist SR144528 (1 mg/kg, i.p., daily), CB2 agonist GW405833 (1 mg/kg, i.p., daily), and PPARγ antagonist GW9662 (1 mg/kg, i.p., daily) were administered 30 min before BCP. The animals were euthanized on protocol day 15.

Single-dose protocol: This protocol was designed to evaluate whether BCP anti-allodynic effects occur shortly after a single dose or depend upon the long-term modulation of target tissues/cells. We took advantage of the therapeutic protocol to minimize the use of experimental animals in an independent experiment. After 3 doses, OXA induced consistent decreases in mechanical thresholds. On protocol day 6, before the first dose of BCP or CB2/PPAR agonists/antagonists, von Frey tests were performed, BCP (or GW405833) was administered, and the animals were re-evaluated for mechanical responses after 1, 3, 6, and 12 h (Figure 1A). When used, SR144528 and GW9662 were administered 30 min before BCP.

### 2.6. Von Frey Test

Mechanical allodynia was evaluated using the von Frey filaments, following the “up and down” method [42]. The test consists of applying von Frey filaments on the hind paw plantar region (footpad area), and removal, shaking, or licking of the stimulated paw is considered a positive response. The animals were placed in the von Frey apparatus for 30 min until they were calm, and then, the test was performed. Six applications were performed, using filaments (Semmes–Weinstein monofilaments) with forces ranging from 0.02 to 4.0 g. The 50% paw withdrawal response threshold was calculated [43] and log-transformed. Unless otherwise specified, von Frey tests were always performed before OXA/BCP administration.

### 2.7. Cold Plate Test

Thermal nociception to cold stimuli was evaluated using an adapted cold plate test [8,9,44]. Unrestrained animals were placed onto a cold plate (22 cm × 12 cm, length × width) with a surface temperature of 4 ± 1 °C, and the number of brisk lift/withdrawal, licking, or shaking reflexes of all paws was recorded for 1 min. The results were expressed as “Paw withdrawal (events/min)”. Unless otherwise specified, cold plate tests were always performed before the OXA/BCP treatment for a given day.

### 2.8. Hargreaves Test

Nociceptive responses to heat were assessed as described by Hargreaves et al. [45]. The test measures time to paw withdrawal after thermal stimulation of the hind paw plantar surface. Heat stimulus was generated using the Hargreaves apparatus (Ugo Basile, Gemonio, Italy). The infrared light intensity was set at 35 (IR: 35). At this light intensity, the animals usually respond within 8–12 s. We used a cut-off time of 20 s to avoid the risk of heat-induced damage. On the day of the experiment, the animals were acclimatized for 20 to 30 min in the Hargreaves apparatus before testing. The tests were performed before the OXA/BCP treatment for a given day. Data were expressed as “Latency to paw withdrawal (seconds)”.

### 2.9. Tissue Harvesting and Sample Processing

The animals were euthanized via intraperitoneal injection of a mixture of ketamine and xylazine, followed by cardiac puncture, on protocol day 15 (i.e., 24 h after the last OXA and BCP administration). The blood was collected via cardiac puncture, transferred to tubes containing 70 μL of 0.1 M EDTA (pH 8.0), and kept at room temperature. The hematological analyses were performed using a BC-2800Vet hematological counter (Mindray Anim. Med. Tech., Shenzhen, China) by Citovet laboratory (Florianópolis, Santa Catarina, Brazil). Tumors were dissected and weighed. Spinal cord samples were collected via hydraulic extrusion. For the TBARS assay, spinal cords were homogenized using Turrax X-1020 apparatus with 1 mL of PBS (pH 7.4) and centrifuged at 3000 rpm for 10 min at 4 °C, and the supernatant was stored at −80 °C. For ELISA, spinal cord samples were homogenized in RIPA (radioimmunoprecipitation assay) buffer containing 1 mM PMSF and protease inhibitor cocktail (#P8340, Sigma-Aldrich, St. Louis, MO, USA) and clarified via centrifugation at 10,000× *g* for 10 min at 4 °C, and the supernatant was stored at −80 °C. The proteins were quantified using the Lowry method [46].

### 2.10. TBARS Assay

Lipid peroxidation was assessed via the thiobarbituric-acid-reactive substances (TBARS) method [47]. Spinal cord lysates (1.5 to 2 mg protein/sample) were incubated with 10% trichloroacetic acid (TCA) to precipitate proteins and centrifuged at 10,000 rpm for 15 min. The supernatants were incubated with 0.67% thiobarbituric acid (TBA) for 40 min in a heat bath, producing a pink-colored Schiff base, which was read at 532 nm using a spectrophotometer (Infinite M200; Tecan, Männedorf, Switzerland). TBARS levels were expressed as nmol/mg of protein.

### 2.11. Enzyme-Linked Immunosorbent Assay (ELISA)

The quantification of interleukin 1beta (IL-1β) and TNF was performed using the mouse IL-1beta/IL-1F2 Quantikine ELISA kit (MLB00C, R&D Systems, Inc., Minneapolis, MN, USA) and Tumor Necrosis Factor-α ELISA Kit (RAB0477, Sigma-Aldrich, St. Louis, MO, USA), respectively, following the manufacturer’s instructions. Mouse IL-10 DuoSet ELISA (DY417, R&D Systems) and Mouse IL-6 DuoSet ELISA (DY406, R&D Systems) were used for IL-10 and IL-6 quantification, respectively. Spinal cord lysates (200 μg protein) in RIPA buffer were used in the sample incubation step. Cytokine concentrations were calculated using the standard curve and expressed as pg of cytokine/mg protein.

For the detection of 4-hydroxynonenal (4-HNE)- modified proteins, we used an indirect ELISA protocol [48]. Briefly, 96-well High Binding Standard ELISA Microplates (Greiner Bio-One, Frickenhausen, Germany) were coated with 100 μL of spinal cord lysates containing 400 μg protein for 6 h, and then, washed three times with washing buffer (RABWASH4, Sigma-Aldrich, St. Louis, MO, USA). Subsequently, the samples were incubated with 200 μL of anti-4-HNE antibody (Ab46545, Abcam, Cambridge, UK; 1:1000) for 2 h at room temperature. Then, the plates were washed three times, and incubated with anti-rabbit IgG peroxidase-linked antibody (1:2000) for 1 h at room temperature. After three washing steps, 100 μL of TMB substrate (Sigma-Aldrich, St. Louis, MO, USA) was added to each well and incubated for 10 min at room temperature. The reaction was stopped with HCl 2N, and absorbance was read at 450 and 540 nm (Infinite M200, Tecan, Männedorf, Switzerland). The results were expressed as absorbance at 450 minus 540 nm (A_450–540 nm_).

### 2.12. DCF Assay

For the ex vivo analysis of ROS production, we used short-term spinal cord slices cultures adapted from a previous publication [49]. Immediately after euthanasia, the thoracolumbar region of the spinal cord was isolated, placed in 3% agarose, and sliced into 500 μm sections using a tissue chopper (Campden Instruments Ltd., Leicestershire, UK). Adjacent spinal cord sections were incubated in 24-well cell culture plates containing 500 μL DMEM supplemented with 1× antibiotic/antimycotic solution (Sigma-Aldrich) for 30 min on ice to wash out cell debris. Subsequently, the medium containing cell debris was discarded and replaced with a fresh medium containing 10 μM of DCFH-DA (dichlorofluorescein diacetate) for ROS detection. The spinal cord sections were incubated for an additional 30 min at 37 °C and 5% CO_2_ in a humidified incubator. The slices were collected, washed in ice-cold PBS (pH = 7.4), manually homogenized in a tissue grinder in the dark, and centrifuged to remove debris, and the fluorescence was read at 485/532 nm (excitation/emission) using a Spectramax Paradigm microplate reader (Molecular Devices, San Jose, CA, USA). The data were expressed as DCF fluorescence units per mg of protein per well. Hydrogen peroxide-treated slices were used as a positive control.

### 2.13. Statistical Analysis

The data are expressed as mean ± standard error of the mean (mean ± SEM). One-way ANOVA followed by Tukey’s post hoc test was used to compare differences between 3 or more groups. Differences among three or more groups under two different conditions (e.g., time and treatment) were analyzed using repeated measures two-way ANOVA with Greenhouse–Geisser correction followed by Tukey’s post hoc test. A significance level of *p* < 0.05 was considered statistically significant. We obtained sample sizes of *n* = 6 or 7 in some biochemical assays instead of *n* = 8/group. This occurred due to either an insufficient protein amount in the spinal cord homogenates for ELISA and TBARS or poor quality of the spinal cord slices for the ex vivo evaluation of the ROS production assay. In addition, TNF levels in the control group were below the assay sensitivity cut-off; even so, they were used for statistical comparison between groups. Statistical analysis was performed in GraphPad Prism^®^ 9.0.0 software. Graphs were created using GraphPad Prism^®^, and figures were assembled in Photoshop (Adobe, San Jose, CA, USA). Graphical abstract was created using BioRender.com (accessed on 17 August 2023).

## 3. Results

### 3.1. Preventive and Therapeutic Intervention with BCP Suppresses Mechanical and Cold Allodynia in OXA-Treated Mice

Initially, we evaluated the dose–response effect of BCP administered concomitantly with OXA from protocol day 0 onwards. In this preventive model, BCP inhibited nociceptive responses to both mechanical (Figure 1B) and cold (Figure 1C) stimuli elicited by OXA. The BCP effect was statistically significant from protocol days 5 to 6 (i.e., after 3 doses of OXA) and at BCP doses of 50 to 250 mg/kg of BCP (Figure 1B,C). The 10 mg/kg dose of BCP exerted only minor impact on mechanical response thresholds and no significant effect on the augmented cold sensitivity caused by OXA exposure (Figure 1B,C). In addition, neither OXA nor BCP changed the latency to paw withdrawal in response to heat (Figure 1D). We also monitored the impact of BCP on the antitumor activity and hematological toxicity of OXA in the preventive model. The results showed that BCP neither improved nor impaired OXA anticancer activity in the Ehrlich model (Figure 1E), and had no benefit in preventing the immunosuppressive effects of OXA on blood leukocytes (Figure 1F).

**Figure 2:**
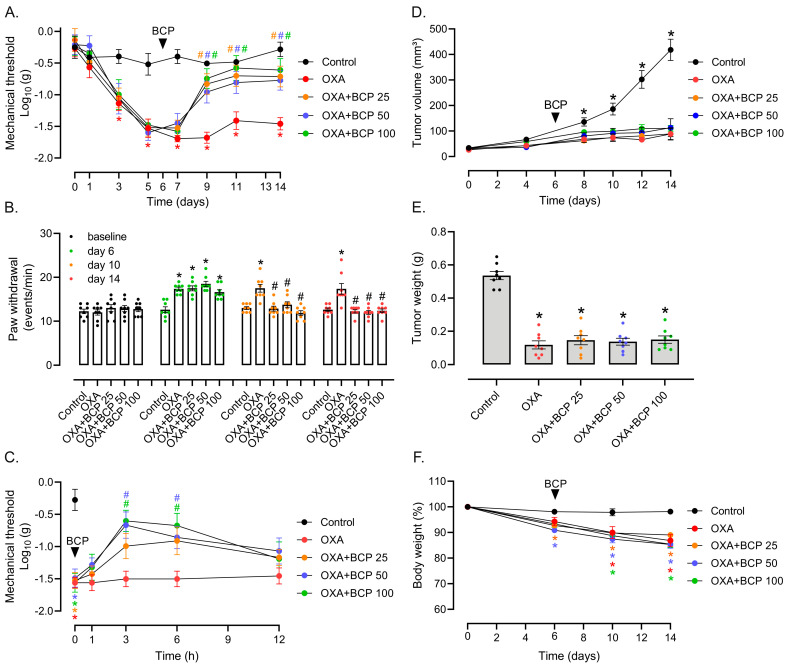
BCP inhibits OXA-induced mechanical and cold allodynia in the therapeutic protocol. (**A**) von Frey and (**B**) cold plate tests in Ehrlich tumor-implanted mice treated with OXA combined with different doses of BCP (25, 50, and 100 mg/kg, 1×/day, gavage) in the therapeutic scheme. Baseline thresholds were taken on protocol day −1, OXA treatment started on day 0, and BCP started on day 6 (detailed in Figure 1A and Section 2). (**C**) Short-term effect (1, 3, 6, and 12 h) of a single dose of BCP (gavage) on mechanical allodynia in tumor-bearing mice with established OIPN. Using the therapeutic protocol, on day 6, mechanical nociception was monitored using the von Frey test after the first administration of different doses of BCP. (**D**) Tumor volume/growth kinetics, (**E**) tumor weight in grams at the end of the experiment, and (**F**) body weight change in tumor-implanted mice treated with OXA + BCP combination in the therapeutic scheme. Body weight per mouse was expressed as a percentage compared to respective weight on protocol day 0. Legend: Control (vehicle-treated); OXA (oxaliplatin); BCP “X” (β-caryophyllene; “X” represents the dose in mg/kg). * Different from the vehicle/control group in (**A**–**C**,**E**,**F**), and different from all other groups in (**D**), considering the same experimental time point; ^#^ different from the OXA group at the same experimental time point (ANOVA; *p* < 0.05; *n* = 8/group).

We next went on to evaluate whether BCP could improve nociceptive responses when given to animals with established peripheral neuropathy, which is more comparable to clinical settings. In this therapeutic intervention, BCP treatment started after sensitivity thresholds had been consistently altered by OXA, which occurred after three doses (protocol days 5 to 6), as assessed via the von Frey (Figure 2A) and cold plate (Figure 2B) tests. BCP at 25, 50, and 100 mg/kg ameliorated both mechanical (Figure 2A) and cold (Figure 2B) allodynia. The BCF effect started after the third/fourth administration (i.e., protocol day 9 for von Frey and protocol day 10 for cold plate tests) (Figure 2B,C), and sustained until the end of the protocol without a dose-dependent pattern (Figure 2A,B). We also took advantage of this therapeutic protocol to investigate the acute effect of a single administration of BCP. On protocol day 6, following the first BCP administration, the mechanical thresholds increased after 3 h treatment with 50 and 100 mg/kg, and remained higher than the OXA group at 6 h with 100 mg/kg of BCP (Figure 2C). However, this single-dose effect was transient and was not sustained until later time points (12 h). This result may help to explain why BCP requires multiple doses to achieve pharmacological efficacy in the therapeutic setting used herein.

In keeping with the results from the preventive model, BCP treatment did not affect Ehrlich tumor growth inhibition caused by OXA (Figure 2D,E). In addition, OXA toxicity to normal tissues also induces body weight loss [9], which was not altered by BCP treatment (Figure 2F). Taken together, the results thus far demonstrate that BCP exhibits an anti-allodynic effect on cold and mechanical allodynia when used in both preventive and therapeutic treatment strategies. Although lower doses (10 and 25 mg/kg) promoted some benefit, higher doses (≥50 mg/kg) showed a more consistent response in the single-dose drug schemes.

### 3.2. CB2 Receptor Mediates BCP Anti-Nociceptive Effects in OXA-Induced Peripheral Neuropathy

**Figure 3:**
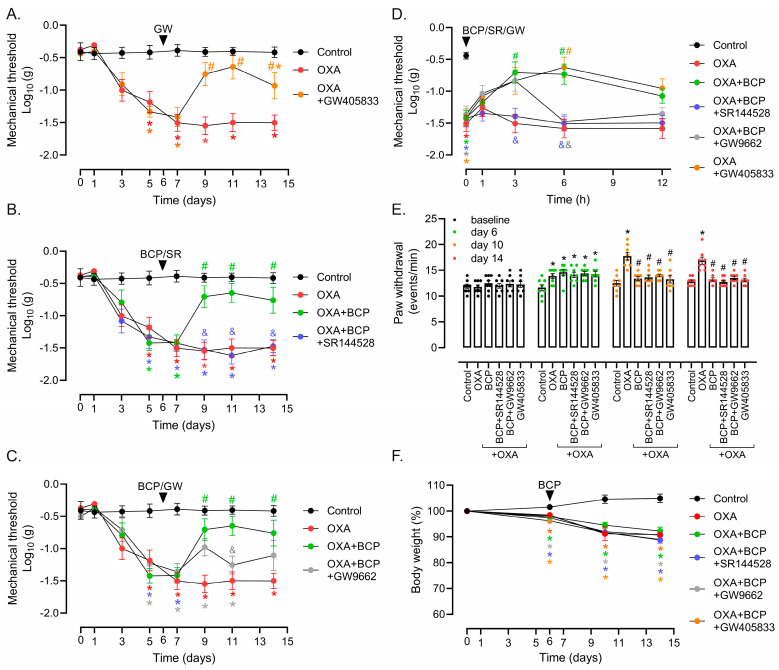
Role of CB2 receptor and PPARγ in BCP anti-nociceptive effect. von Frey tests showing the effect of the (**A**) CB2 agonist GW405833 (1mg/kg; i.p. daily), (**B**) BCP combined with the CB2 receptor antagonist SR144528 (1 mg/kg, i.p. daily), and (**C**) BCP combined with the PPARγ antagonist GW9662 (1 mg/kg, i.p. daily) in the therapeutic protocol. OXA treatment started on day 0, and BCP (100 mg/kg, gavage, daily) with or without CB2 and PPAR antagonists started on protocol day 6 (see Figure 1A and Section 2 for details). Data shown in panels (**A**–**C**) were collected from the same experiment, and split to ease visualization of the tested agonist/antagonists. Note that the “control” and “OXA” groups’ data are the same in the (**A**–**C**) panels. (**D**) Short-term effect (1, 3, 6, and 12 h) of a single dose of BCP (100 mg/kg, gavage) combined with SR144528 (1 mg/kg, i.p. daily) or GW9662 (1 mg/kg, i.p. daily) in animals with established neuropathy caused by OXA. The effect of the CB2 agonist GW405833 (1 mg/kg; i.p. daily) in OXA-treated mice is also shown. Using the therapeutic scheme, on protocol day 6, mechanical nociception was monitored using the von Frey test after the first administration of BCP with/without antagonists. (**E**) Cold plate test showing the effect of CB2 and PPARγ antagonists combined with BCP, and of the CB2 agonist GW405833, upon cold sensitivity at baseline (protocol day −1) and different time-points in the therapeutic model. (**F**) Body weight changes in mice treated with OXA/BCP in the presence or absence of CB2 and PPARγ antagonists. Body weight per mouse was expressed as a percentage compared to respective weight on protocol day 0. * Different from the vehicle/control group, ^#^ different from the OXA group, and ^&^ different from the OXA + BCP group at the same experimental time point (ANOVA; *p* < 0.05; *n* = 8/group).

We next determined the contribution of the CB2 receptor upon the BCP anti-allodynic effect. Moreover, it has been described that the pharmacological effects of BCP may involve PPARγ in vascular inflammation, arthritis, and colitis models [31,32,34]. In the therapeutic model, the selective CB2 agonist GW405833 (1 mg/kg, i.p., daily) reversed OXA-induced mechanical allodynia, with statistical significance from protocol days 9 to 14 (Figure 3A). Corroborating these results, the CB2 antagonist SR144528 (1 mg/kg, i.p., daily) inhibited the anti-allodynic effect of BCP (100 mg/kg, gavage, 1×/day) in the von Frey experiments (Figure 3B). We also examined whether the PPARγ antagonist GW9662 could hinder BCP activity in the OXA model. GW9662 caused a transient blockage of the BCP anti-allodynic response at the dose tested (1 mg/kg, i.p., daily), which was statistically significant only on protocol day 11 in the von Frey test (Figure 3C). However, the short-term analysis of the mechanical responses following a single dose of BCP combined with SR144528 and GW9662 revealed that both antagonists were able to block, at least in part, BCP efficacy after the 3 and/or 6 h treatment in animals with established neuropathy (Figure 3D). Intriguingly, neither SR144528 nor GW9662 were capable of inhibiting BCP’s anti-allodynic effect in the cold plate test (Figure 3E). Even though this result indicates that CB2-independent mechanisms play a role in the BCP control of cold allodynia, the activation of CB2 by a selective agonist, GW405833, decreased OXA-induced cold hypersensitivity to levels comparable to the control group (Figure 3E). As a control, none of the agonists/antagonists tested altered body weight loss related to OXA treatment (Figure 3F). Of note, the tumor implant step of the therapeutic protocol was not performed in the Figure 3 experiments. Therefore, the data thus far suggest that the CB2 receptor is indeed a potential target for pharmacological intervention in OXA-induced neuropathy, controlling both mechanical and cold sensitivity. Regarding BCP, while its effects on mechanical stimuli involve the CB2 receptor, the control of cold allodynia seems to depend on other mechanisms.

### 3.3. BCP Decreases ROS Production, Oxidative Damage, and Inflammatory Cytokine Levels in the Spinal Cords of OXA-Treated Mice

Anti-inflammatory and antioxidant effects stand out among the pharmacological activities attributed to BCP. We previously demonstrated that different antioxidants ameliorated neuropathy-like phenotypes and decreased inflammatory cytokine levels in the spinal cords of mice treated with OXA [9]. To test whether BCP alters oxidative stress and cytokine parameters in our model, we isolated spinal cord tissues from animals treated with OXA and OXA combined with 100 mg/kg BCP in the therapeutic protocol. The sensitivity tests reproduced the previous results of BCP upon mechanical and cold allodynia, as well as the non-effect of BCP upon OXA antitumor activity (Supplementary Figure S1). In this cohort, we also established a control group without tumor implants, which displayed sensitivity thresholds similar to the tumor-implanted control, thereby suggesting that the treatment of OXA largely determines the outcome (i.e., neuropathy-like phenotype) in our model. In contrast, the contribution of subcutaneous, small Ehrlich tumors generated in the model seems minor.

**Figure 4:**
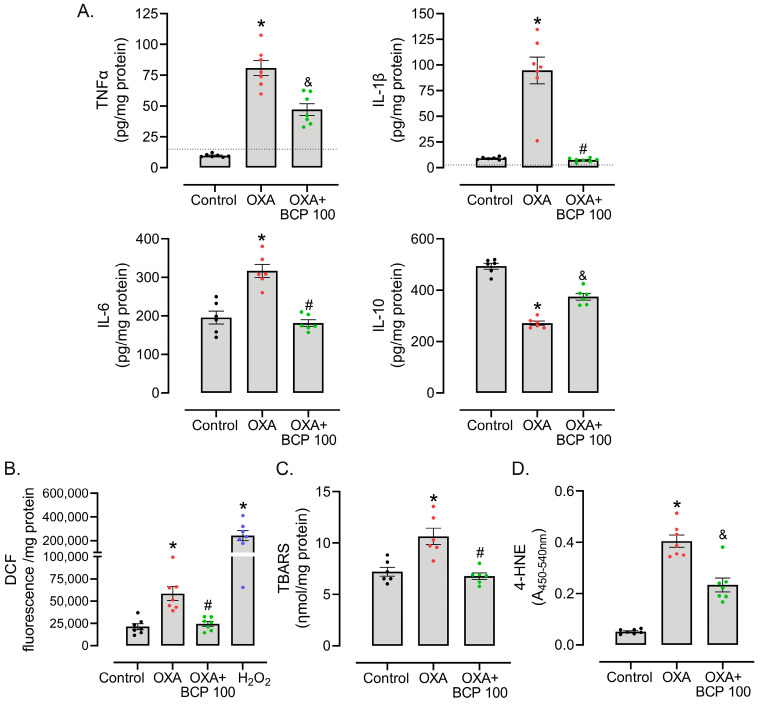
BCP suppresses OXA-induced inflammatory cytokine production and spinal cord oxidative damage. (**A**) ELISA assay quantification of TNF, IL-1β, IL-10, and IL-6 levels, (**B**) ex vivo DCF assay, (**C**) TBARS, and (**D**) 4-HNE content in the spinal cords of tumor-bearing animals treated with OXA with/without BCP (100 mg/kg, gavage, daily) (*n* = 6–7/group). OXA treatment started on day 0, BCP (100 mg/kg, gavage, daily) started on day 6, and spinal cord tissues were isolated on protocol day 15 for cytokine and oxidative stress evaluation. In (**A**), the dashed line denotes the assay sensitivity cut-off. * Different from vehicle/control group, ^#^ different from OXA group, and ^&^ different from both OXA and control groups, considering the same experimental time point (ANOVA; *p* < 0.05).

Regarding inflammatory mediators, OXA-treated mice showed an increased level of TNF, IL-1β, and IL-6, and decreased content of the anti-inflammatory cytokine IL-10, as determined by ELISA, in spinal cord lysates from protocol day 15 (Figure 4A). Animals treated with BCP showed IL-1β, and IL-6 levels similar to the control group, while TNF and IL-10 levels were partially restored (Figure 4A). These anti-inflammatory effects exerted by BCP accompany decreased ROS production in the spinal cord when compared to OXA-treated mice, as inferred from the DCF assay (Figure 4B). In addition, OXA-induced ROS seems to be associated with oxidative damage to biomolecules, with an augmented content of TBA-reactive lipoperoxidation products (Figure 4C) and protein adducts of 4-HNE, an aldehyde formed during lipoperoxidation (Figure 4D), in OXA-treated mice. BCP abrogated, at least in part, spinal cord TBARS and 4-HNE protein adduct formation in animals treated with OXA (Figure 4D).

In contrast to the pharmacological effects in the spinal cord, BCP did not improve the hematological toxicity of OXA, as determined by means of leucocyte (control: 6.1 + 1.9; OXA: 2.1 + 0.7 *; OXA + BCP: 2.8 + 0.9, cells ×10^3^/μL *n* = 6, * *p* < 0.05, ANOVA–Tukey), hematocrit (control: 42.3 + 3.5%; OXA: 31.6 + 3.1% *; OXA + BCP: 32.8 + 1.2% *, *n* = 6, * *p* < 0.05, ANOVA–Tukey), and hemoglobin (control: 13.7 + 1.1 g/dL; OXA: 10.7 + 0.9 g/dL *; OXA + BCP: 10.6 + 0.2 g/dL *; *n* = 6, * *p* < 0.05, ANOVA–Tukey) quantification in blood samples.

## 4. Discussion

The management of OIPN remains a challenge in clinical practice, mostly due to a lack of highly effective therapies. Herein, we report that the CB2 receptor agonist BCP has pharmacological potential to treat OIPN, with mechanisms involving CB2 receptors as expected, as well as regulating inflammatory and redox homeostasis in the spinal cord. Importantly, most of the experiments were performed in tumor-bearing mice, which allowed us to demonstrate that BCP did not impair the effects of OXA on tumor development. Regarding nociceptive responses, BCP prevented mechanical and cold allodynia when administered at the onset of OXA therapy, as well as reversed an established OIPN phenotype in the therapeutic protocol. The benefits of BCP administered post induction of OIPN, as well as the positive results in the short-term assays with a single dose, indicate that BCP interferes with mechanisms controlling the transmission/sensitization of nociceptive signals, and not directly by blocking OXA genotoxicity to peripheral neurons. The lack of effect of BCP against OXA-induced leucopenia and tumor growth inhibition suggests that the OXA mechanism of action via platinum–DNA adduct formation is likely preserved.

To our knowledge, this study constitutes the first report on the efficacy of BCP upon OXA-induced neuropathy. Regarding the chemotherapy-induced neuropathy models, our results are in line with the decreased allodynia induced by paclitaxel in BCP-treated animals [39]. In a study using the synthetic CB2 agonist AM1710, the authors demonstrated decreased responses to mechanical and cold stimuli in a neuropathy model induced by paclitaxel and cisplatin [50]. In addition, the CB2 agonist JHW-133 improved mechanical nociception in a cisplatin-induced neuropathy model [41]. In a paclitaxel-induced neuropathic pain model, the mixed CB1/CB2 agonist CP55,940 [51] and CB2 receptor agonist MDA7 [52] improved mechanical allodynia. In our model, CB2 receptors participate, at least in part, in the mechanism of action of BCP, given that the CB2 antagonist SR144528 abrogated the beneficial effects of BCP upon mechanical sensitivity in animals treated with OXA. Moreover, the results with the selective CB2 agonist GW405538 support the role of CB2 receptor activation in reducing the OIPN phenotype. Thus, the data from our group and others indicate that CB2 agonists are a promising strategy for the treatment of peripheral neuropathy caused by OXA, with a potential broader impact on neuropathies caused by other anticancer drugs.

In the context of OIPN, there is growing evidence that pro-inflammatory cytokines are produced in key sites along the nociceptive pathway, such as the spinal cord and dorsal root ganglion [9,18,19,20,21]. In rats, Janes et al.’s study found that OXA increased the expression of TNF and IL-1β, but decreased IL-10 and IL-4, in the dorsal horn of the spinal cord [19]. In addition to the spinal cord, OXA augments TNF, IL-1β, IL-6, and CXCL12, and decreases IL-10 in the dorsal root ganglion after 10 days of treatment in rats [18]. In keeping with these data, we observed an augmented content of IL-1β, IL-6, and TNF, and reduced levels of IL-10 protein, thereby indicating a pro-inflammatory state in the spinal cord of animals treated with OXA. Moreover, diverse pharmacological interventions that decrease pro-inflammatory cytokines in OXA-induced neuropathy also exert anti-nociceptive effects in animal models [9,19,20,21]. These results build upon the theory that neuroinflammation occurs as a result of OXA exposure and could be targeted to treat OIPN symptoms.

CB2 receptor activation is associated with the downregulation of inflammatory cytokines in different models, including neuroinflammation [22,27,52,53]. For instance, the CB2 agonist MDA7 prevented mechanical hyperalgesia and reduced the expression of TNF, IL-1β, and IL-6 in the spinal cords of mice with neuropathy induced by paclitaxel [52]. In a carrageenan-induced paw inflammation model, the CB2 agonist GW405833 decreased serum concentrations of TNF and IL-1β [27]. In addition, treatment with the CB2 agonist JHW133 reduced brain levels of TNF and IL-1β in a model of acoustic stress in mice [53]. In our model, BCP restored the spinal cord inflammatory state (i.e., the cytokine profile) to levels, at least in part, similar to the control group. Corroborating these results, BCP decreased IL-1β and NF-κB levels in the spinal cords of paclitaxel-treated mice [39]. Furthermore, several studies in different models have demonstrated the anti-inflammatory actions of BCP. In a model of dyslipidemia induced by a high-fat diet in rats, BCP treatment resulted in reduced TNF and IL-1β levels in the aorta [32]. In a multiple sclerosis model, Alberti et al. (2017) reported that BCP reduced IFN-γ and increased IL-10 production in T cells treated with MOG35-55 peptide in vitro, and prevented microglial activation and demyelination in C57BL/6 mice in vivo [54]. In a model of doxorubicin-induced cardiotoxicity in rats, BCP reduced inflammatory cytokines, oxidative stress, and cell death markers in the heart in a CB2-dependent manner [55]. Therefore, BCP and other CB2 agonists exert anti-inflammatory effects in different pathologies, including chemotherapy-induced neuropathy. Whether CB2 receptors fully mediate the anti-inflammatory effects of BCP, or whether other pathways also play a role in this phenotype, requires further investigation.

Our results also demonstrated that OXA increased lipoperoxidation, ROS production, and 4-HNE levels, thereby characterizing an unbalanced redox environment in the spinal cord that has damaged biomolecules. BCP ameliorated these parameters, possibly indicating potential neuroprotective activity involving antioxidant mechanisms. Even though the role of BCP in controlling redox homeostasis in OXA-induced neuropathy is novel, evidence from other models suggests that BCP can activate the NRF2 pathway, which is an important transcription factor that controls antioxidant and xenobiotic detoxification [35,36,37,54,56]. We previously reported that compounds with antioxidant properties (i.e., N-acetylcysteine, vitamin E, and α-lipoic acid) reduce spinal cord oxidative stress and inflammation, and inhibit OIPN, in Swiss mice [9]. OXA is well-described as an inducer of ROS production and DNA damage, including DNA platination in nociceptive neurons [8,9,12,13,15,16,17]. In this regard, it has been reported that OXA elicits dislocation of Zonula Occludens-1, one of the tight junction proteins that constitute the BBB [57], possibly explaining the accumulation of OXA in the brains of rats [13]. The increased permeability of the BBB allows the chemotherapy agent to enter the brain parenchyma, affecting both the neuronal and glial compartments [58,59]. Experiments carried out in sciatic nerves and spinal cords obtained from OXA-treated animals strengthened the role of ROS and oxidative damage in the onset of neuropathic pain [8,9]. Nociceptive receptors such as TRPA1 are redox-sensitive, activated by ROS, and associated with OXA-induced neuropathic pain [7,60]. Likewise, ROS may promote tissue damage, recruiting peripheral and/or activating tissue-resident immune cells, leading to inflammatory responses and generating a positive feedback loop between inflammation and oxidative stress. In our model, BCP attenuated both inflammation and oxidative stress in the spinal cord. However, we identified neither the CB2-expressing cell type nor the nociceptive terminal/site targeted by BCP in OXA-treated mice. For example, depending upon the pain model, CB2 receptor activation in the periphery (i.e., paw/intraplantar) [33,61], dorsal root ganglion, and spinal cord [62] may exert an anti-nociceptive effect in inflammatory and neuropathic pain models. Therefore, it is conceivable that CB2-dependent and -independent mechanisms—such as the antioxidant effect observed herein—may contribute to the anti-OIPN activity of BCP. The CB2-independent regulation of nociceptive pathways may help to explain why the CB2 antagonist did not intervene with the anti-allodynic effect of BCP upon cold sensitivity, at least at the doses tested in our model.

Finally, assessing whether drug candidates for OIPN therapy affect tumor development is of utmost importance. In this context, BCP did not affect the antitumor effectiveness of OXA on Ehrlich carcinoma in either preventive or therapeutic schemes. Hanušová et al. (2017) demonstrated that BCP did not influence doxorubicin antitumor activity in mice with Ehrlich tumor implants [63]. On the other hand, CB2 agonists are potential drug candidates in some cancer types. For instance, the CB2 agonist JHW-133 reduced tumor volume and lung metastasis, and the CB2 antagonist SR144528 abrogated this effect, in breast cancer xenografts [64]. Even though it cancers are notorious for being highly heterogeneous at the level of genetic alterations and tumor microenvironment composition, which could lead to cancer-type-dependent responses, these data suggest that BCP is unlikely to hamper the anticancer activity of OXA.

This study has limitations. Firstly, the anti-neuropathic effect of BCP was determined only in female mice with OIPN. Even though it has been described that BCP promotes antinociception in paclitaxel-treated male mice [39], prior studies in inflammatory pain [65] and spinal cord injury [66] reported that BCP is able to decrease pain behaviors in both sexes, but to a greater degree in males. Therefore, possible sexual dimorphism of BCP pharmacological activity (i.e., sex-related differences in the magnitude of BCP anti-neuropathic effects) in the OIPN model remains to be tested. In addition, we obtained results from a single mouse strain (Swiss mice, outbred) and cancer type (Ehrlich tumors), and lacked comparisons with other drugs used to treat neuropathic pain such as duloxetine and pregabalin. Therefore, the anti-neuropathic and tumor growth-related effects of BCP require further testing and validation in other animal models of cancer and chemotherapy-induced neuropathy.

## 5. Conclusions

In summary, the results presented herein show that BCP ameliorates OXA-induced mechanical and cold allodynia in both preventive and therapeutic drug treatment schemes in a mouse model of OIPN. The effects of BCP on OXA-induced mechanical sensitivity involve CB2 receptors, whereas a CB2-independent mechanism seems to mediate BCP activity in cold allodynia. BCP also promotes antioxidant and anti-inflammatory responses by decreasing OXA-induced pro-inflammatory cytokine levels, ROS production, and oxidative damage in the spinal cord. Finally, BCP affects neither the antitumor activity nor hematological toxicity of OXA. Taken together, these results indicate BCP as a promising compound for further investigation in OIPN treatment.

## Data Availability

The data are contained within the article and the Supplementary Materials.

## Supplementary Materials

The following supporting information can be downloaded at: https://www.mdpi.com/article/10.3390/antiox12101893/s1, Figure S1: Supplementary to Figure 4.
