# Supplementary material for: β-Caryophyllene Inhibits Oxaliplatin-Induced Peripheral Neuropathy in Mice: Role of Cannabinoid Type 2 Receptors, Oxidative Stress and Neuroinflammation
Source: Antioxidants (Basel). 2023 Oct 22;12(10):1893. doi: 10.3390/antiox12101893 (PMC10604080; doi:10.3390/antiox12101893)
Supplement: Supplementary file 1 [file antioxidants-12-01893-s001.zip › antioxidants-2637598-supplementary.pdf]

Agnes et al. (2023)
Supplementary Figure S1:

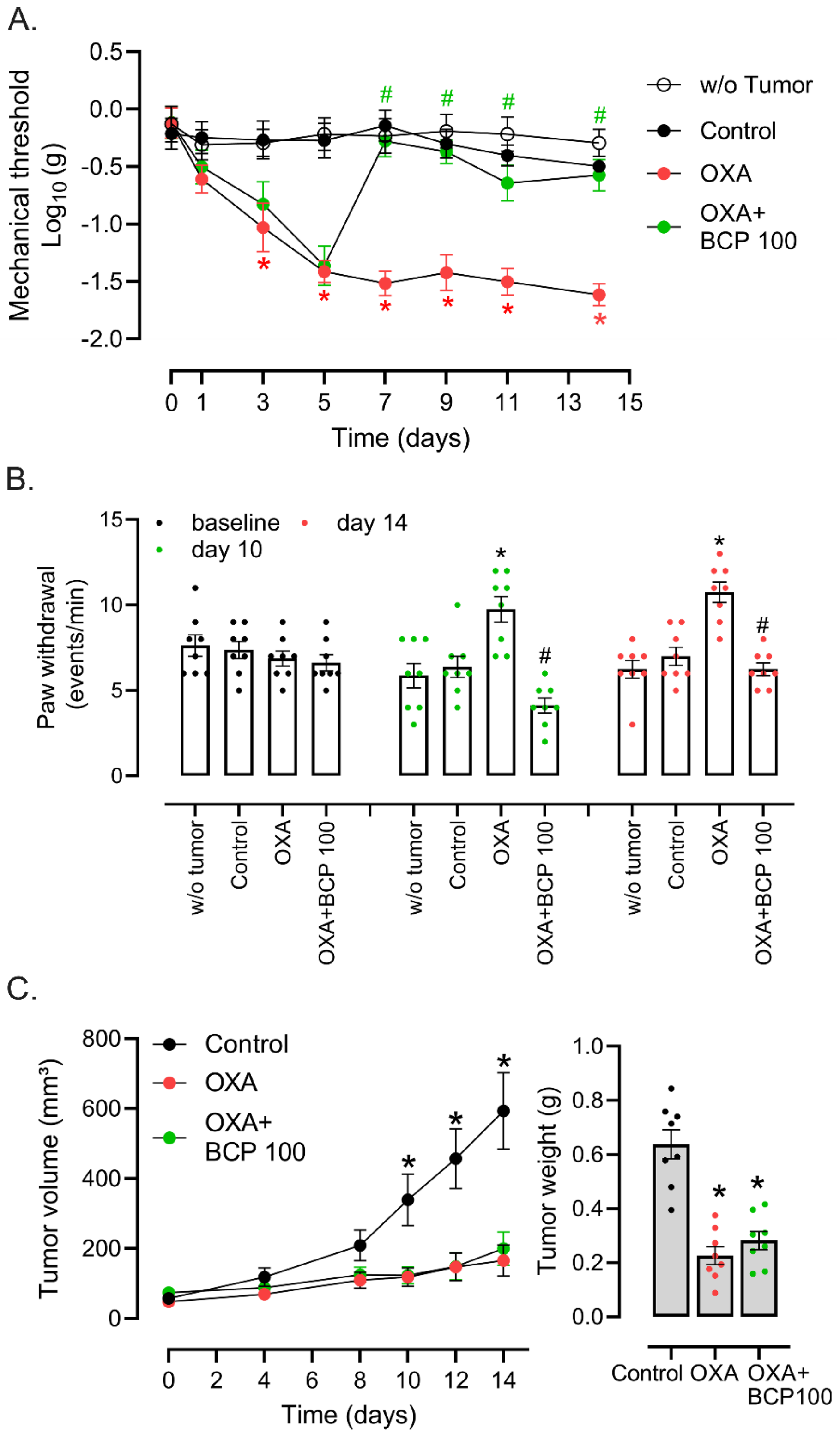

Figure S1: Supplementary to figure 4. (A) Von Frey, (B) cold plate, (C) tumor volume and weight in the therapeutic protocol. In this experiment, tumor-free animals ("w/o tumors" group) were also examined, showing that the presence of tumors did not affect basal nociceptive thresholds in the model (n=8/group). * Different from vehicle/control and "w/o tumor" groups in A and B, *different from all other groups in C, and #different from the OXA group, considering the same experimental time point (ANOVA; $p < 0.05$).
